# The effect of music therapy on hemodialysis patients' quality of life and depression symptoms

**DOI:** 10.1590/2175-8239-JBN-2018-0023

**Published:** 2018-09-13

**Authors:** Paula de Marchi Scarpin Hagemann, Luis Cuadrado Martin, Carmen Maria Bueno Neme

**Affiliations:** 1 Universidade Estadual Paulista Júlio de Mesquita Filho, Faculdade de Ciências, Bauru, SP, Brasil.; 2 Universidade Estadual Paulista Júlio de Mesquita Filho, Departamento de Clínica Médica, Botucatu, SP, Brasil; 3 Universidade Estadual Paulista Júlio de Mesquita Filho, Departamento de Psicologia, Bauru, SP, Brasil.

**Keywords:** Music Therapy, Renal Dialysis, Quality of Life, Depression, Musicoterapia, Diálise Renal, Qualidade de Vida, Depressão

## Abstract

**Introduction:**

The sources of stress involved in chronic kidney disease (CKD) can lead to depressive states, directly affecting the hemodialysis patient's quality of life (QOL). There are few reports and studies on therapeutic interventions that aim to minimize depressive symptoms in these patients and an even greater shortage of studies using music therapy.

**Objective:**

This study evaluated the effect of music therapy on QOL and depressive symptoms in hemodialysis patients.

**Method:**

This was a music therapy intervention study in which 23 patients were evaluated regarding QOL and depression symptoms at two distinct stages - before and after the intervention. Eight sessions of music therapy were conducted, with two weekly sessions and an average duration of 75 minutes. The intervention was conducted by the music therapist herself, using specific music therapy techniques, besides voice and guitar to conduct harmonic and rhythmic support for the groups' sound-music production.

**Results:**

The patients showed a significant reduction in depression symptoms (p < 0.001) and better QOL results, with significant differences in the following dimensions: functional capacity (p = 0.011), pain (p = 0.036), general health (p = 0.01), vitality (p = 0.004), mental health (p = 0.012), symptom and problem list (p = 0.01), and overall health (p = 0.01).

**Conclusions:**

Intervention with music therapy constitutes an effective option in the treatment and prevention of depressive symptoms and improved QOL of HD patients.

## RESUMO

**Introdução:**

Fontes de estresse representadas pela doença renal crônica (DRC) podem gerar estados de depressão, interferindo diretamente na qualidade de vida (QV) dos pacientes em hemodiálise (HD). Poucos são os relatos e estudos de intervenção terapêutica visando minimizar os sintomas depressivos nesses pacientes, com maior escassez de trabalhos utilizando a musicoterapia.

**Objetivo:**

O presente estudo avaliou o efeito da musicoterapia na QV e nos sintomas depressivos em pacientes em HD.

**Método:**

Estudo de intervenção musicoterapêutica, no qual 23 pacientes foram avaliados quanto à QV e sintomas de depressão em duas fases distintas: pré e pós-intervenção. Foram realizadas duas sessões em grupo por semana, com duração de 75 minutos, em um período de quatro semanas. A intervenção foi realizada por um musicoterapeuta, que utilizou técnicas específicas da musicoterapia, além da voz e do violão para conduzir apoio rítmico e harmônico na produção sonoro-musical dos grupos.

**Resultados:**

Os pacientes apresentaram redução significativa dos sintomas de depressão (p<0,001) e melhores resultados na QV, com diferenças estatísticas significantes nas dimensões: capacidade funcional (p=0,011), dor (p=0,036), estado geral de saúde (p=0,01), vitalidade (p=0,004), saúde mental (p=0,012), lista de sintomas e problemas (p=0,01) e saúde global (p=0,01).

**Conclusões:**

A intervenção com musicoterapia constitui-se opção efetiva no tratamento e prevenção de sintomas depressivos e na melhora da QV de pacientes em HD.

## INTRODUCTION

Chronic kidney disease (CKD) is characterized by a slow, progressive, and irreversible loss of kidney function, and in its most advanced stage, kidney replacement therapy is indicated. Treatment options include artificial blood purification methods: hemodialysis (HD) and peritoneal dialysis.^1^^,^ 2 In Brazil, 92.1% of terminal chronic kidney patients undergo HD.^3^

Despite the quantitative increase in CKD patient survival due to the technological and therapeutic advances in recent decades,^4^ frequently this progress is not accompanied by a significant recovery of physical, emotional, and social conditions.^5^ The diagnosis and treatment of CKD can result in a significant impact and numerous losses and limitations in the patient's daily life, leading to various biopsychosocial changes.^4^^,^ 6 All of these changes, together with the limitations of CKD itself, can have a significant effect on the patient's quality of life (QOL). For the dialytic CKD patient, QOL can be understood as the individual's perception of well-being, ranging from satisfaction to dissatisfaction, in relation to areas of life that are important to him.^7^

Some complications of advanced CKD,^8^ as anemia and mineral bone disorders, associated with HD impose extensive changes on the patient's routine, which can lead to a negative effect on health-related QOL, increasing daily stress and favoring the emergence of depression.^4^^,^ 9 ^,^ 10 This, in turn, directly affects the patient's QOL, changing how the individual perceives and evaluates his life and his illness, which may cause him to not adhere to treatment. A patient's negative perception of his own health is associated more with anxiety and depression than with disease severity.^4^^,^ 11 The presence of depression symptoms and anxiety in HD patients negatively affects QOL^12^ and is associated with other impairments, such as cognitive impairments.^13^

Depression is a common and underdiagnosed problem and an independent risk factor for increased morbidity and mortality in these patients.^14^ Given the above, there is a need to evaluate QOL and depression symptoms in HD patients and to intervene therapeutically to facilitate transformation that is consistent with each patient's situation and to prevent a compromise of his daily activities.^9^^,^ 14

The literature has shown the therapeutic effect of music on the physical and emotional well-being of HD patients.^15^^,^ 16 In general, these studies are conducted by nursing teams, and the proposed intervention is based on listening to music, either recorded or played live during HD sessions. A systematic review and meta-analysis published in 2015^17^ found that musical intervention effectively reduced anxiety symptoms in HD patients. Despite the positive results, the authors noted that seven of the included studies used only recorded songs rather than live performances. Furthermore, none of the studies mentioned an intervention by a music therapist.

Listening to music can provide moments of unwinding and relaxation and can therefore have therapeutic effects. All activities involving music are likely to have therapeutic effects, but only music therapy as a science and technology has explicit therapeutic goals, and this is the only area of knowledge that uses music for therapeutic purposes.^18^ To differentiate the use of music by a music therapist from that by other professionals, Dileo^19^ distinguished *music therapy in medicine* from *music in medicine*. The former is performed by qualified music therapists, using specific music therapy methods and techniques. It always involves a therapeutic process, and the relationship will develop from the music and from the process. *Music in medicine*, on the other hand, is performed by health professionals in general as a complementary therapy in various situations (stress, anxiety, pain). There is no therapeutic process, and the relationship between the patient and these professionals is not based on the musical activity.

Music therapy has been studied and applied in various areas of the hospital context^20^^,^ 21 and has proven to be an effective therapy for the treatment of depression.^22^ With regard to its use in HD, few studies have been found,^23^ with none relating exclusively to QOL and depression symptoms. A study conducted in South Korea^24^ showed the contribution of music therapy in reducing anxiety and depression symptoms, leading the authors to suggest music therapy as an intervention that can contribute to an improved QOL in HD patients.

Despite the potential of music therapy to improve QOL and depression symptoms in patients undergoing invasive and risky treatments such as HD, intervention studies are required. Therefore, the objective of this study was to evaluate QOL and depression symptoms in HD patients, both before and after the music therapy process.

## METHOD

This was a music therapy intervention study conducted with 23 HD patients in the *Centro de Terapia Renal Substitutiva/Diálise - CTRS* of the *Hospital Estadual de Bauru.* The patients met the following inclusion criteria: being 18 years of age or older and having terminal CKD and undergoing HD treatment for a period of three months or more. This study excluded patients who were under psychotherapeutic care, initiated and/or changed the use or dosage of psychotropic drugs in the month before the intervention, were participating in other intervention studies, had a diagnosis of dementia, had a hearing or severe mental impairment, were clinically unstable, and were in daily HD patients. Of the 89 patients approached between November 2013 and June 2014, 30 were excluded, resulting in a sample of 59 patients, of whom 31 agreed to participate in the study. Of this total, two dropped out before the start of the intervention, three experienced a significant worsening of clinical symptoms, one underwent transplant, and two patients were unable to form a group, resulting in a sample of 23 patients.

### PROCEDURES

The study was approved by the Research Ethics Committee of the *Faculdade de Ciências de Bauru* at *Universidade Estadual Paulista - UNESP* (Protocol 956,333). Only HD patients who expressed a voluntary desire to participate in the study and who signed the Terms of Free and Informed Consent participated.

The music therapy sessions were conducted with groups of four participants. All patients were evaluated at two distinct stages - before (Stage 1) and after (Stage 2) the music therapy process. At Stage 1, all participants were evaluated for depression symptoms and QOL. The evaluations were conducted by the researcher during HD sessions, always during the first hour. After these evaluations, the participants went through an individual interview during the HD session, in which sociodemographic and clinical data were collected. A music therapy clinical file was also used, which was formulated based on the assumptions established by Barcellos;^23^ these state the need for the music therapist to know the patient's sound history and to outline the therapeutic goals. After the initial evaluations, the music therapy sessions commenced. Eight sessions of music therapy were conducted with each of the six groups, with two weekly sessions and an average duration of 75 minutes per session. The music therapy sessions were conducted by the music therapist researcher herself, always during the first half of the HD session.

The music therapy techniques described by Keneth Bruscia^25^ were used to conduct the sessions: musical recreation, musical improvisation, musical composition, and listening to music or receptive experience. These techniques were used according to the needs of each group, with flexible prior planning. During the sessions, the music therapist researcher used voice and guitar to conduct harmonic and rhythmic support for the groups' sound-music production. In all sessions, the participants had at their disposal speakers, songs in mp3 format (managed by the researcher), and various percussion instruments.

At the end of the music therapy process, all participants responded again to the depression symptoms and QOL instruments (Stage 2).

### INSTRUMENTS

#### SOCIODEMOGRAPHIC AND CLINICAL DATA FORM

This form was developed specifically for this study to obtain data that would allow the participant to be characterized with respect to sociodemographic aspects (gender, age, education, occupation, civil status, children, race, and religion) and clinical medical record data (underlying disease, time on treatment, comorbidities, vascular access, and laboratory exams).

#### DEPRESSION SYMPTOMS INSTRUMENT

The Beck Depression Inventory - Second Edition (BDI-II) was chosen to evaluate depression symptoms. This instrument was originally created by Beck, Ward, Mendelson, Mock, and Erbaugh in 1961 and reviewed by Beck, Rush, Shaw, and Emery in 1979/1982.^26^ The BDI-II is a self-administered instrument comprising 21 items, each of which has four statements relating to content with increasing degrees of depression severity, with scores ranging from zero to three. The Brazilian version of the second edition was developed by Gorenstein et al.^27^ The depression intensity classification, according to the BDI-II score, is as follows: minimum - 0-13; mild - 14 to 19; moderate - 20-28; and severe - 29-63. In this study, scores ≥ 14 points were considered to indicate the presence of depressive symptoms.

#### QUALITY OF LIFE INSTRUMENT

The instrument chosen to evaluate HRQOL was the *Kidney Disease and Quality of Life Short Form* (KDQOL-SF) in its translated version, adapted and validated for Brazilian culture.^28^ The dimensions of the KDQOL-SF(tm) are measured on a standardized scale ranging from zero (worst score - unfavorable HRQOL) to 100 (best score - favorable HRQOL), with higher scores indicating better health. This scale is considered easy to understand and administer. The instrument includes the *Short-Form Health Survey* (SF-36) as a generic measure that evaluates eight domains of the patient's physical and mental health. In addition, to address the CKD patient, 12 specific dimensions are included with respect to the disease.

#### STATISTICAL ANALYSIS

Initially, a descriptive analysis with data consistency check was performed. The prevalence of depressive symptoms and the mean score obtained on the BDI-II, in addition to the means obtained in the different QOL domains according to the KDQOL-SF, were calculated with the respective standard deviations. Data are presented as the mean±standard deviation or median (first and third quartile), where appropriate.

The t-test for paired samples (for data with a normal distribution) and the Wilcoxon test (for data with a non-normal distribution) were used to compare the data collected at stages 1 and 2, and the chi-squared test was used for categorical variables. In addition to these comparisons, correlations were examined between the KDQOL-SF dimensions, the score obtained on the BDI-II, and the demographic variables, using Pearson's correlation coefficient. P values < 0.05 were considered significant in all analyses. SPSS 12.0 was the statistical program used for data storage and analysis.

## RESULTS

The intervention was performed with 23 patients; the sociodemographic and clinical characteristics are shown in Table 1. It should be noted that none of the participants had changes of vascular access during the study and all of them had a Kt/V greater then 1.2. The variation of serum levels of hemoglobin, calcium, and phosphorus was not significant during the Stages of the study.

**Table 1:** Patient sociodemographic and clinical data

Variable	%
Gender (N = 23)	
Female	56.5
Male	43.5
Age group (N = 23) (54.9 ± 14.6)	
18-39 years	26.1
40-59 years	26.1
60 years or older	47.8
Race	
White	69.5
Black	30.5
Civil status (N = 23)	
Married	69.5
Separated	21.7
Single	4.3
Widowed	4.3
Education (N = 23) (7.1 ± 4.1)	
1-4 years	39.1
5-8 years	26.1
High School	26.1
Higher Education	8.7
Occupation (N = 23)	
Retired	34.7
Unemployed	4.3
Sick leave	43.4
Stay-at-home spouse	8.6
Retired and working	8.6
Family income (N = 23)	
< 1 minimum wage	4.3
Up to 1 minimum wage	13
Up to 3 minimum wages	47.8
Up to 5 minimum wages	13
Over 5 minimum wages	21.7
Time on treatment (N = 23) (31.5 ± 18.6)	
0-6 months	4.3
7-36 months	60.8
37-60 months	26.1
61 months or more	8.7
Underyling disease	
Hypertension	13
*Diabetes mellitus*	56.5
Chronic glomerulonephritis	21.7
Other	8.6
Main comorbidities	
*Diabetes Mellitus*	60.8
Cardiovascular disease	13
Hypertension	78.2
Smoking	8.6
Vascular access	
Fistula	65.2
Catheter	34.7

At Stage 1 of the study, a prevalence of 60.8% (n = 14) of depressive symptoms was found. Among these cases, 34.7% (n = 8) were classified as having mild symptoms, 13% (n = 3) moderate symptoms, and 13% (n = 3) severe symptoms. After the music therapy intervention, the prevalence of depressive symptoms was reduced to 21.7% (n = 5), with 17.3% (n = 4) being classified as mild symptoms, and 4.34% (n = 1) as moderate symptoms. A comparison of the prevalence of depressive symptom revealed that the improvement in symptom intensity was significant (p = 0.017), as shown in Table 2. When these results were analyzed as continuous variables, the depressive symptoms' score reduced from 15.43 ± 9.2 at Stage 1 to 7.43 ± 6.4 at Stage 2 (p < 0.001).

**Table 2:** BDI-II according to depression symptom intensity, categorized into case and non-case

Evaluations	BDI-II				
	Case		Non-case		p
	N	%	N	%	
Stage 1	14	60.8	9	39.1	0.017
Stage 2	5	21.7	18	78.26	

BDI-II: Beck Depression Inventory.

The KDQOL-SF questionnaire is divided into generic and specific dimensions, and the results for these are shown in Table 3. Regarding the comparison of mean KDQOL-SF generic dimension scores attributed by patients in the evaluations before and after the intervention, there was significance for functional capacity (p = 0.011), pain (p = 0.036), general health status (p = 0.01), vitality (p = 0.004), and mental health (p = 0.012). In the specific dimensions, significance was found for symptom and problem list (p = 0.01) and overall health (p = 0.01).

**Table 3:** Comparison between patient scores on quality of life dimensions (KDQOL-SF) before and after music therapy intervention

Dimensions (number of items)	Stage 1	Stage 2	P
Generic			
Functional Capacity	60.0 ± 24.77	66.0 ± 23.6	**0.011**
Physical aspects*	50 (25; 100)	75 (25; 100)	0.497
Pain	65.6 ± 25.7	78.2 ± 23.9	**0.036**
General health status	54.3 ± 27.3	68.0 ± 24.9	**0.010**
Vitality	63.0 ± 21.6	73.2 ± 20.1	**0.004**
Social aspects*	75 (53; 100)	87 (75; 96)	0.208
Emotional aspects	78.2 ± 31.1	81.1 ± 33.0	0.732
Mental health	64.5 ± 24.5	76.8 ± 20.1	**0.012**
Specific			
Symptoms and problems list	80.6 ± 12.6	85.6 ± 11.6	**0.010**
Effects of kidney disease	72.4 ± 16.3	77.1 ± 19.4	0.064
Kidney disease burden	46.4 ± 29.7	54.3 ± 34.3	0.197
Professional role*	0 (0; 0)	0 (0; 37.5)	1.0
Cognitive function	79.4 ± 18.8	85.5 ± 15.6	0.130
Quality of social interaction	75.9 ± 23.5	76.2 ± 20.9	0.941
Sexual function**	90.9 ± 11.3	92.7 ± 14.1	0.569
Sleep	67.8 ± 19.0	70.3 ± 18.8	0.373
Social support*	100 (67; 100)	100 (87; 100)	0.339
Encouragement from dialysis team	86.4 ± 18.0	86.9 ± 22.4	0.915
Overall health*	70 (50; 80)	80 (62; 82)	**0.010**
Patient satisfaction	66.6 ± 15.8	67.5 ± 14.4	0.815

Values are expressed as the mean±standard deviation or median (first, third quartile). Stage 1: Before music therapy intervention; Stage 2: after music therapy intervention.

* Wilcoxon test;

** Sexual Function: 12 sexually active patients at Stage 1 and 13 sexually active patients at Stage 2.

Because gender differences regarding the studied variables - QOL and depression symptoms - can occur, the results of males and females were compared, with no significant difference between them being found for any of the evaluated variables.

Regarding the correlations between data obtained from the BDI-II and the KDQOL-SF, which are presented in Table 4, it is important to note that in Stage 1, the BDI-II depression symptoms score - although a continuous variable - negatively correlated with several QOL dimensions, both generic and specific, with special attention to general health (*r =* -0.664; p = 0.001), social aspects (*r =* -0.611; p = 0.002), and mental health (*r =*-0.619; p = 0.002).

**Table 4:** Correlation between the BDI-II and generic (SF-36) and specific quality of life dimensions

		BDI-II	FC	PA	PAIN	GHS	VIT	SA	EA	MH
BDI-II	r		-0.324	-0.408	-0.486	-0.664	-0.512	-0.611	0.077	-0.619
	p		0.131	0.053	**0.019**	**0.001**	**0.012**	**0.002**	0.729	**0.002**
LSP	r	-0.573	0.432	0.447	0.602	0.688	0.696	0.678	-0.059	0.591
	p	**0.004**	**0.040**	**0.032**	**0.002**	**0.000**	**0.000**	**0.000**	0.788	**0.003**
EKD	r	-0.621	0.368	0.219	0.468	0.640	0.574	0.595	0.210	0.581
	p	**0.002**	0.084	0.316	**0.024**	**0.001**	**0.004**	**0.003**	0.336	**0.004**
KDB	r	-0.455	0.341	0.464	0.289	0.329	0.616	0.239	-0.035	0.375
	p	**0.029**	0.112	**0.026**	0.181	0.126	**0.002**	0.272	0.872	0.078
PR	r	-0.306	0.085	-0.048	0.061	0.227	0.181	0.110	0.262	0.400
	p	0.155	0.700	0.829	0.783	0.297	0.408	0.617	0.227	0.059
FC	r	-0.415	0.232	0.355	0.668	0.449	0.571	0.520	0.213	0.544
	p	**0.045**	0.287	0.096	**0.000**	**0.032**	**0.004**	**0.011**	0.330	**0.007**
QSI	r	-0.473	0.337	0.469	0.390	0.591	0.713	0.626	0.177	0.790
	p	**0.023**	0.116	**0.024**	0.066	**0.003**	**0.000**	**0.001**	0.419	**0.000**
SXF	r	-0.524	0.154	0.254	0.245	0.411	0.670	0.678	0.099	0.609
	p	0.080	0.632	0.426	0.444	0.184	**0.017**	**0.015**	0.759	**0.036**
SLP	r	-0.525	0.039	0.299	0.275	0.247	0.439	0.316	-0.153	0.292
	p	**0.010**	0.861	0.166	0.204	0.255	**0.036**	0.142	0.484	0.76
SCS	r	-0.509	0.197	0.495	0.342	0.487	0.541	0.692	0.002	0.696
	p	**0.013**	0.367	**0.016**	0.110	**0.018**	**0.008**	**0.000**	0.992	**0.000**
TS	r	-0.208	0.140	-0.074	0.084	0.275	0.147	0.082	-0.146	0.155
	p	0.342	0.523	0.737	0.704	0.205	0.504	0.708	0.505	0.481
OH	r	-0.392	0.331	0.267	0.204	0.466	0.576	0.529	0.038	0.552
	p	0.064	0.123	0.218	0.351	**0.025**	**0.004**	**0.009**	0.863	**0.006**
PS	r	-0.594	0.096	0.065	0.111	0.463	0.341	0.567	0.051	0.403
	p	**0.003**	0.662	0.767	0.614	**0.026**	0.111	**0.005**	0.817	0.056

BDI-II: Beck Depression Inventory. Abbreviations of generic quality of life dimensions: FC = functional capacity; PA = physical aspects; PAIN = pain; GHS = general health status; VIT = vitality; SA = social aspects; EA = emotional aspects; MH = mental health. Abbreviations of specific quality of life dimensions: LSP = List of symptoms and problems; = EKD effects of kidney disease; KDB = kidney disease burden; PR = professional role; FC = cognitive function; QSI = quality of social interaction; SXF = sexual function; SLP = sleep; SCS = social support; TS = team stimulus; OH = overall health; PS = patient satisfaction.

Correlations between the generic QOL dimensions and age and time on treatment variables were also observed. In regard to the age variable, it was noted that it negatively correlated with functional capacity (*r* = -0.450, p = 0.031). The time on treatment negatively correlated with the generic dimensions general health status (*r* = -0.563; p = 0.005), vitality (*r* = -0.563; p = 0.005), and mental health (*r* = - 0.548; p = 0.007).

In regard to specific QOL dimensions, it was noted that the age variable negatively correlated with sexual function (*r* = -0.719; p = 0.008) and that time on treatment negatively correlated with cognitive function (*r* = -0.413; p = 0.050) and quality of social interaction (*r* = -0.487, p = 0.018).

## DISCUSSION

The results of this study indicate that music therapy has a beneficial effect in reducing depressive symptoms and improving the QOL of the studied population. To the best of our knowledge, this research constitutes the first study to evaluate the effect of music therapy on HD patients' QOL and depression symptoms. It was evident that music therapy caused significant differences between the stages before and after intervention, improving QOL and reducing the intensity of patients' depressive symptoms. There is evidence in the literature regarding the need to intervene and promote changes in the daily lives of these patients to improve their QOL, and music therapy is an option.^9^^,^ 14

In regard to depression, it was found that the prevalence of depressive symptoms at Stage 1 was 60.8%. These indices were higher than those of similar studies in the literature, which found depressive symptom prevalence of 42.7%^29^ and 33.3%.^30^ Both of these studies were Brazilian and also used the BDI to evaluate depression symptoms. The high rates of depressive symptoms found in this study reveal a concerning situation, particularly considering that patients were not included in the study because they already had disorders, including depression, and that some refused to participate, which could further increase this prevalence, notwithstanding. Due to the great severity of their clinical status, HD patients are more likely to suffer from depression than the general population. Depression symptoms in terminal CKD patients should be diagnosed and treated because these patients are significantly more likely to commit suicide than people in the general population.^31^

A comparison of the depression symptoms before and after the intervention confirms the hypothesis that guided this study, that music therapy sessions would improve these patients' depression symptoms. The benefits of music therapy in the treatment of depression do not occur randomly. The change agent for depressive symptoms is the "active doing" that occurs in the therapeutic relationship between the music therapist and the patient, playing musical instruments and singing.^32^ This must be the reason why randomized music therapy trials show high levels of engagement with patient groups with whom is traditionally difficult to engage.^22^^,^ 32

In the present study, the participants had impaired health statuses, as demonstrated by their low scores in some QOL domains. With respect to the generic dimensions (SF-36), the ones related to physical aspects had the worst scores at Stage 1 of the study, which corroborates the data found in the literature.^9^ In regard to the evaluation of specific QOL dimensions in this study, the most affected were those related to kidney disease burden and the professional role, which corroborated the existing data.^33^^-^ 35 The low score obtained for the professional role may be explained by the observation that a significant portion of the studied sample (43.4%) had not retired and depended on social health care benefits to survive.

Comparing the results obtained at stages 1 and 2 of this study, it can be observed that the participants showed a significant improvement in both generic QOL dimensions and specific CKD dimensions. The improvement in QOL across all of these dimensions may be explained by the playful character of music, by the active participation promoted by the production of rhythms and melodies, and by the group integration that occurs in music therapy sessions. A study conducted in northern Taiwan shows that listening to music during HD can promote the patient's general well-being. The use of music can thus serve as a complementary treatment.^36^ In addition, the exchange and sharing of experiences among HD patients may lead to establishing positive links between them and thus help cope with the treatment routine.

Correlating generic (SF-36) and specific (KDQOL-SF) QOL dimensions revealed that symptom and problem list and kidney disease burden substantially affected patients' QOL. Because of the effect that CKD has on QOL, patients need psychological and emotional support.^35^ With regard to the negative correlations between the BDI-II results and various QOL dimensions, there is evidence in the literature that depression symptoms and QOL dimensions have an inversely linear relationship.^33^

The main limitation of this study is the absence of a control group. As the interventions occurred during all the periods of HD, even patients not participating in the study could have positive effects indirectly. Another limitation involves the instruments used. Although BID-II is the gold standard instrument for identifying depressive symptoms, it is not specific to HD patients. Regarding the KDQOL-SF, although it is a QOL instrument specific to an HD population, its evaluation is subjective. Therefore, it is suggested that further research be conducted with these and other instruments to further clarify our results.

Because of the positive results achieved in this study, it is emphasized that similar surveys should be conducted with a greater number of participants, with more time allocated to the music therapy intervention, and with the use of a control group. The strengths of the present study include the fact that it is pioneering research and that, to date, no other study has simultaneously evaluated the effect of music therapy on QOL and depression symptoms in HD patients. Despite the care and restrictions necessary within an HD situation, high patient participation with the music therapy was possible without any incident or injury, as highlighted by the participants and the health team.

Data from the Brazilian Society of Nephrology Census *(Sociedade Brasileira de Nefrologia)* estimated that in 2016, approximately 122,825 patients were on dialysis. Thus, there is a growing need to evaluate emotional factors and QOL and take actions to minimize issues that may affect the patient's routine, treatment, and adherence to treatment.
